# Inhibitory Effect of Cinobufagin on L-Type Ca^2+^ Currents, Contractility, and Ca^2+^ Homeostasis of Isolated Adult Rat Ventricular Myocytes

**DOI:** 10.1155/2014/496705

**Published:** 2014-05-25

**Authors:** Pinya Li, Qiongtao Song, Tao Liu, Zhonglin Wu, Xi Chu, Xuan Zhang, Ying Zhang, Yonggang Gao, Jianping Zhang, Li Chu

**Affiliations:** ^1^Hebei Medical University, 361 East Zhongshan Road, Shijiazhuang, Hebei 050017, China; ^2^The Fourth Hospital of Hebei Medical University, Shijiazhuang, Hebei 050011, China; ^3^Department of Pharmacology, Hebei University of Chinese Medicine, Shijiazhuang, Hebei 050200, China

## Abstract

Cinobufagin (CBG), a major bioactive ingredient of the bufanolide steroid compounds of Chan Su, has been widely used to treat coronary heart disease. At present, the effect of CBG on the L-type Ca^2+^ current (*I*_Ca-L_) of ventricular myocytes remains undefined. The aim of the present study was to characterize the effect of CBG on intracellular Ca^2+^ ([Ca^2+^]_i_) handling and cell contractility in rat ventricular myocytes. CBG was investigated by determining its influence on *I*_Ca-L_, Ca^2+^ transient, and contractility in rat ventricular myocytes using the whole-cell patch-clamp technique and video-based edge-detection and dual-excitation fluorescence photomultiplier systems. The dose of CBG (10^−8^ M) decreased the maximal inhibition of CBG by 47.93%. CBG reduced *I*_Ca-L_ in a concentration-dependent manner with an IC_50_ of 4 × 10^−10^ M, upshifted the current-voltage curve of *I*_Ca-L_, and shifted the activation and inactivation curves of *I*_Ca-L_ leftward. Moreover, CBG diminished the amplitude of the cell shortening and Ca^2+^ transients with a decrease in the time to peak (Tp) and the time to 50% of the baseline (Tr). CBG inhibited L-type Ca^2+^ channels, and reduced [Ca^2+^]_i_ and contractility in adult rat ventricular myocytes. These findings contribute to the understanding of the cardioprotective efficacy of CBG.

## 1. Introduction

**Figure 1:**
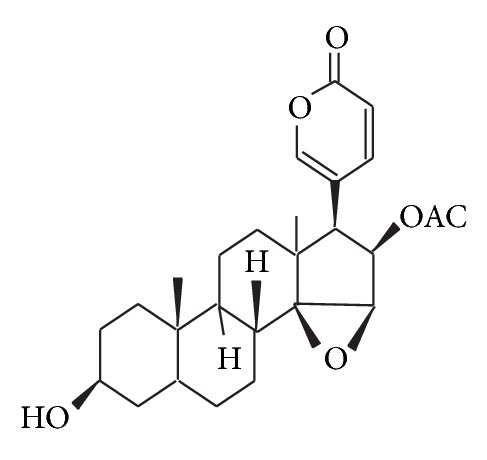
Structure of CBG.

Chan Su, a traditional Chinese medicine prepared from the dried white secretion of auricular glands and skin glands of Chinese toads (*Bufo melanostictus* Schneider or* Bufo bufo gargarizans* Cantor) [1], has attracted the attention of many clinicians due to its diverse biological activities, such as cardiotonic and antitumor [2, 3]. The chemical structure of CBG (C_26_H_34_O_6_), the main active ingredient isolated from Chan Su, has been determined (Figure 1). Chan Su is not only used as a traditional Chinese medicine, but it is also listed in Pharmacopoeia of People's Republic of China (PRC; State Pharmacopoeia Committee, 2010). Extracts of Chan Su, also known as dried toad venom in English, “toad cake” in China, “Senso” in Japan, and “Somsa” in Korea [4, 5], are used in cardiac therapies.

Although the pharmacological profile of Chan Su has been extensively characterized, research on Chan Su has mainly focused on its toxicity. Chan Su in high doses causes cardiac arrhythmia, seizure, and coma. Death of one woman has been reported after the consumption of Chinese herbal tea [6] and of another woman following ingestion of Lu Shen Wan pills containing Chan Su [7]. Therefore, the use of Chan Su by the general population was recommended to be avoided due to its toxicity [8]. Interestingly, formulations of Chan Su have long been widely applied in China, Japan, and other Asian countries and are currently used as a component of other traditional medicines such as Kyushin and Shexiang Baoxin Pill (SBP) [9–11]. Kyushin and SBP have been successfully used for many years in the treatment of numerous ailments, especially coronary artery heart diseases (CHD) such as angina, coronary artery spasm, and myocardial infarction (MI) [12]. In addition, chemical analyses have shown intact CBG and its metabolites in plasma and urine of healthy volunteers receiving a drug Kyushin containing Chan Su by enzyme immunoassay after the separation of these compounds by high performance liquid chromatography (HPLC) [11]. CBG also has been detected in SBP by HPLC [13]. SBP has demonstrated synergistic effects in rats with MI with a therapeutic mechanism based on inhibiting dysfunctions in energy metabolism, oxidative injury, and inflammation in the development of MI [14, 15]. Therefore, the current clinical evidence for these medicines supports the potential cardioprotective effects of CBG.

As a major steroid compound isolated from Chan Su, CBG has been reported by some to exert its pharmacological effects on voltage-gated potassium (K^+^) channels in primary cultures of rat hippocampus neurons [16]. Nevertheless, direct evidence for the effects of CBG on L-type Ca^2+^ channels (LTCC) is lacking. Increased contractility of ventricular myocytes is a well-known central feature of the cardiac response to ischemic myocardial diseases [17]. In pathological conditions such as ischemia, the amount of Ca^2+^ that crosses the sarcolemma exceeds the Ca^2+^ sequestration and extrusion capacity of the cell, leading to the disturbance of the intracellular Ca^2+^ ([Ca^2+^]_i_) homeostasis. This Ca^2+^ overload is characterized by a rise in [Ca^2+^]_i_ to a level that triggers pathological events in the cell, such as arrhythmias, mechanical dysfunction (e.g., increased diastolic tension, reduced peak force, and relaxation rate), and eventually cell death [18]. In addition, the opening of LTCCs is associated with Ca^2+^ overload [19], and LTCC blockers have generally proven to be effective when given during the ischemic period, illustrating their energy sparing properties. Since drugs that suppress cardiac LTCC and contractility, such as Ca^2+^ antagonists and *β*-adrenoceptor blocking agents, are able to effectively protect the myocardium from ischemic injury, we speculated that CBG exerts its cardioprotective effects via inhibition of LTCCs and cardiac contractility. Therefore, we used the whole-cell patch-clamp technique and video-based edge-detection and dual-excitation fluorescence photomultiplier systems in this study to study the influence of CBG on the L-type Ca^2+^ current (*I*_Ca-L_), Ca^2+^ transient, and contractility in rat ventricular myocytes. Additional research on the cellular mechanisms of CBG will not only contribute to a better understanding of the efficacies of Chan Su in clinical treatments, but also provide experimental evidence for rational applications of Kyushin and SBP.

## 2. Materials and Methods

### 2.1. Drugs and Reagents

CBG was obtained from Beijing SLF Chemical Research Institute (Beijing, China). Collagenase type II was obtained from Gibco (GIBCO, Invitrogen, Carlsbad, CA, USA). Bovine serum albumin (BSA), HEPES, and taurine were obtained from Roche (Basel, Switzerland). Verapamil (Ver) was purchased from Harvest Pharmaceutical Co., Ltd. (Shanghai, China). Unless otherwise stated, other chemical reagents were obtained from Sigma Chemical Co. (St. Louis, MO, USA). All solvents used are of analytical purity.

### 2.2. Isolation of Adult Rat Ventricular Myocytes

Adult male Sprague-Dawley rats weighing 220–280 g were purchased from the Experimental Animal Center, Hebei Medical University. All animal handling procedures were in accordance with the Guidelines of Animal Experiments from the Committee of Medical Ethics, National Health Department of China. Briefly, adult rats were injected intraperitoneally with 1,500 IU heparin and anesthetized intramuscularly with an ethyl carbamate (0.8 g/kg). The heart was rapidly excised, cannulated, and perfused retrogradely with Ca^2+^-free Tyrode's solution containing (in mM) NaCl 135, KCl 5.4, MgCl_2_ 1.0, glucose 10, and HEPES 10 (pH 7.4 with NaOH) via the aorta on a Langendorff perfusion apparatus until spontaneous contractions ceased and the efflux was clear (3 min). The heart was perfused with Ca^2+^-free Tyrode's solution containing 4 g/L collagenase type II, 4 g/L taurine, and 10 g/L BSA for 20–30 min until the heart was flaccid. Enzymes were washed out with Krebs buffer (KB) solution containing (in mM) KOH 80, KCL 40, KH_2_PO_4_ 25, L-glutamic acid 50, taurine 20, HEPES 10, EGTA 1, glucose 10, and MgSO_4_ 3 (pH 7.2 with KOH). All solutions used during the perfusion were bubbled with 100% O_2_ and maintained at 37°C. After the perfusion, the ventricles were placed in a beaker filled with KB solution and minced. Ventricular myocytes were dispersed by shaking the beaker gently, and the undigested tissue was removed by filtration through a 200 *μ*m nylon mesh. The cells were kept in KB solution (bubbled with 100% O_2_) at room temperature for at least 1 h. Cells were used within 9 h of isolation.

Experiments with rat ischemic ventricular myocytes were performed according to the method of Wu et al. [20]. Briefly, rats were anesthetized with an ethyl carbamate (0.8 mg/kg); vasopressin (1.5 IU/kg, i.v.) was injected intravenously by tail vein to induce cardiac ischemia. After 10 min of ischemia, the heart was removed and used for experiments as normal rat ventricular myocytes above.

### 2.3. Electrical Recordings

Whole-cell patch recordings were performed on ventricular myocytes sustaining LTCCs. Experiments were performed at room temperature (22–25°C). Pipettes were pulled from borosilicate glass capillaries and had resistances of 2~5 MΩ when filled with the internal solution. Currents were recorded using an Axon patch 200B amplifier and pClamp 10.0 software (Axon Instruments, Union City, CA, USA) and were filtered at 2 KHz. The external solution used to record LTCCs contained (in mM) TeaCl 140, MgCl_2_ 2.0, CaCl_2_ 1.8, glucose 10, HEPES 10 (pH 7.4 with CsOH). The internal solution for perforate patch recording consisted of (in mM) CsCl 20, TeaCl 20, Mg-ATP 5, HEPES 10, and EGTA 10 (pH 7.2 with CsOH).

### 2.4. Measurements of Cell Contractions

Cell shortening of ventricular myocytes was assessed by a video-based edge-detection system (IonOptix, Milton, MA, USA). Briefly, the cells were placed in a perfusion chamber mounted on the stage of an inverted microscope (IonOptix) and perfused with normal Tyrode's solution with 1.8 mM CaCl_2_ at a rate of 1 mL/min. The cells were field stimulated at a frequency of 0.5 Hz (2 msec duration). The myocyte being studied was displayed on the computer monitor with the aid of an IonOptix MyoCam charge-coupled device camera, which was attached to the sidearm of the microscope. Only rod-shaped myocytes with clear edges were selected for experiments. Experiments were performed at room temperature.

### 2.5. Measurements of [Ca^2+^]_i_ Transients

Myocytes were loaded with fura-2/AM for 15 min at room temperature in the dark, and fluorescence measurements were recorded with a dual-excitation fluorescence photomultiplier system (IonOptix). The myocyte being field stimulated at 0.5 Hz was imaged through a Fluor 40x oil objective and exposed to light, which was emitted by a 75 W lamp and passed through a 340 or 380 nm filter (bandwidths were ±15 nm) alternately. The emitted fluorescence was detected at 510 nm (between 480 and 520 nm at both excitation wavelengths). Qualitative changes in [Ca^2+^]_i_ levels were inferred from the ratio of the fluorescence intensity at two wavelengths (340/380). Ca^2+^ transients were calibrated as described previously [21].

### 2.6. Statistics

The activation and steady-state inactivation curves of *I*_Ca-L_ were fitted using Boltzmann functions. The chord conductance was calculated using the ratio of the current to the electromotive force in individual current-voltage (*I-V*) relationships. These conductances were normalized to their individual maximal conductance. Data were expressed as means ± S.E.M. All data were analyzed statistically using one-way analysis of variance (ANOVA) followed by Student's *t*-test. *P* < 0.05 was considered to be statistically significant.

## 3. Results

### 3.1. Confirmation of *I*_Ca-L_

**Figure 2:**
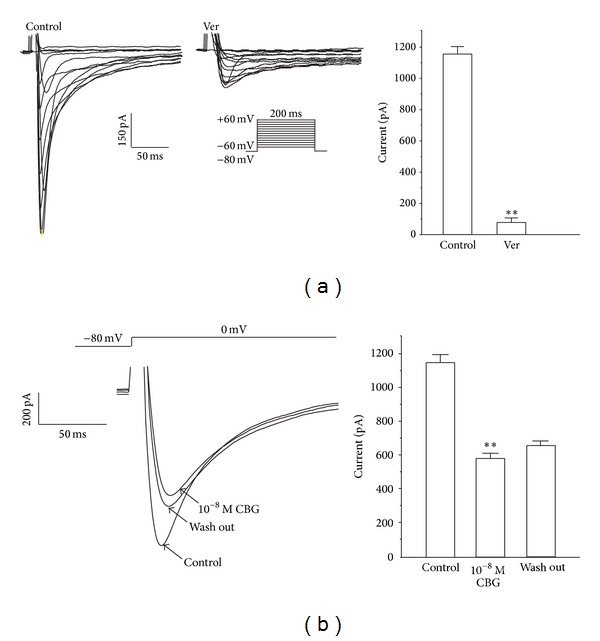
Ver (0.1 mM) completely blocks *I*_Ca-L_ in rat ventricular myocytes. (a) Representative *I*_Ca-L_ recordings according to the steady-state activation protocol before and after application of Ver. Summary data of *I*_Ca-L_ before and after application of Ver. (b) *I*_Ca-L_was recorded under control conditions, during exposure to 10^−8^ M CBG and during wash out. Data are means ± S.E.M. (*n* = 6 cells). **P < 0.01, versus control.

As shown in Figure 2, *I*_Ca-L_ was elicited according to the steady-state activation protocol. Application of Ver (0.1 mM), a specific LTCC blocker, nearly completely blocked *I*_Ca-L_ (Figure 2(a)) (*P* < 0.01), indicating that these currents were Ca^2+^ currents. After washing out CBG with the external solution, the *I*_Ca-L_ partially recovered (Figure 2(b)).

### 3.2. Dose-Dependent Effects of CBG on *I*_Ca-L_

**Figure 3:**
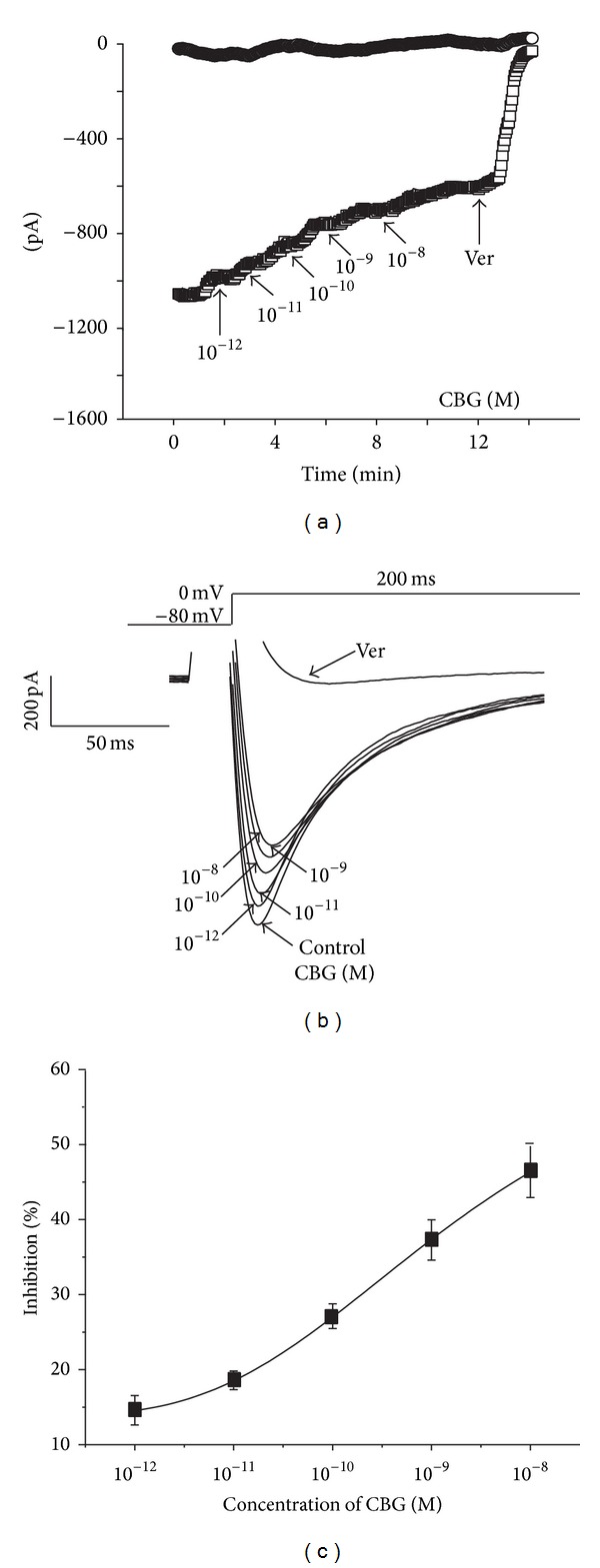
CBG dose-dependently inhibits *I*_Ca-L_. (a) Time course of *I*_Ca-L_ recorded under control conditions, during exposure to 10^−12^, 10^−11^, 10^−10^, 10^−9^, and 10^−8^ M CBG and 0.1 mM Ver. (b) Traces of *I*_Ca-L_ evoked in the absence and presence of CBG derivatives. (c) Dose-response curve demonstrating the inhibitory effects of CBG on *I*_Ca-L_. Data are presented as means ± S.E.M. (*n* = 5–7 cells).

Current traces elicited by depolarization from the test potential of −80 mV to 0 mV at different CBG concentrations are shown in Figure 3(a). *I*_Ca-L_ was progressively suppressed by increasing concentrations of CBG (from 10^−12^ to 10^−8^ M) (Figure 3(b)). The peak amplitude of *I*_Ca-L_ was decreased by 14.81 ± 2.04%, 18.81 ± 0.74%, 27.85 ± 1.35%, 38.46 ± 2.08%, and 47.93 ± 3.58% by CBG derivatives at 10^−12^, 10^−11^, 10^−10^, 10^−9^, and 10^−8^ M, respectively. The time dependency of the CBG effects on *I*_Ca-L_ is shown in Figure 3(c). After exposing the cells to CBG at 10^−12^, 10^−11^, 10^−10^, 10^−9^, and 10^−8^ M, *I*_Ca-L_ decreased by 14.81%, 18.81%, 27.85%, 38.46%, and 47.93%, respectively.

### 3.3. Effects of CBG on* I-V* Relationship of *I*_Ca-L_

**Figure 4:**
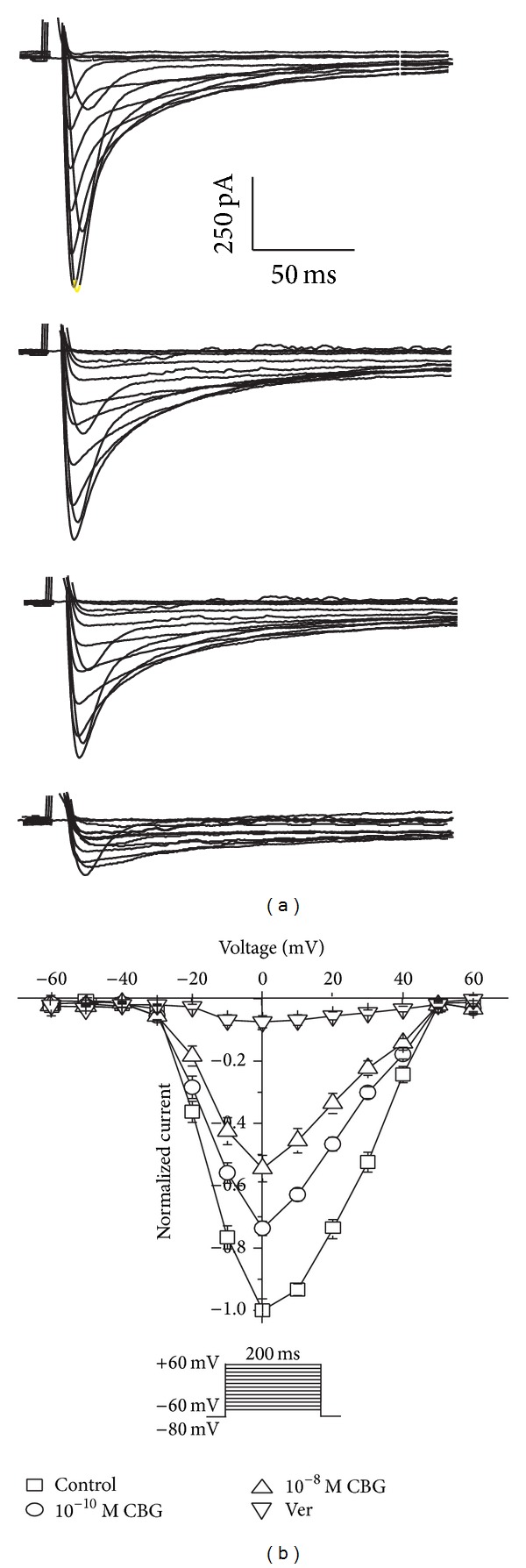
Effects of CBG on* I-V* curve of *I*_Ca-L_. (a)-(b): Exemplary traces (a) and pooled data (b) show effects of CBG at different concentrations on the* I-V* relationship. Data are presented as means ± S.E.M. (*n* = 8 cells).

Representative traces elicited by depolarization from the test potential of −60 mV to 60 mV at different CBG concentrations are shown in Figure 4(a). The* I-V* relationship for Ca^2+^ currents between −60 and 60 mV revealed that the normalized currents decreased from −0.99 ± 0.01 pA to −0.72 ± 0.02 pA, −0.53 ± 0.04 pA, and −0.06 ± 0.01 pA in the presence of 10^−10^ M CBG, 10^−8^ M CBG, and 0.1 mM Ver, respectively (Figure 4(b)).

### 3.4. Effects of CBG on *I*_Ca-L_ of Ischemic Ventricular Myocytes

**Figure 5:**
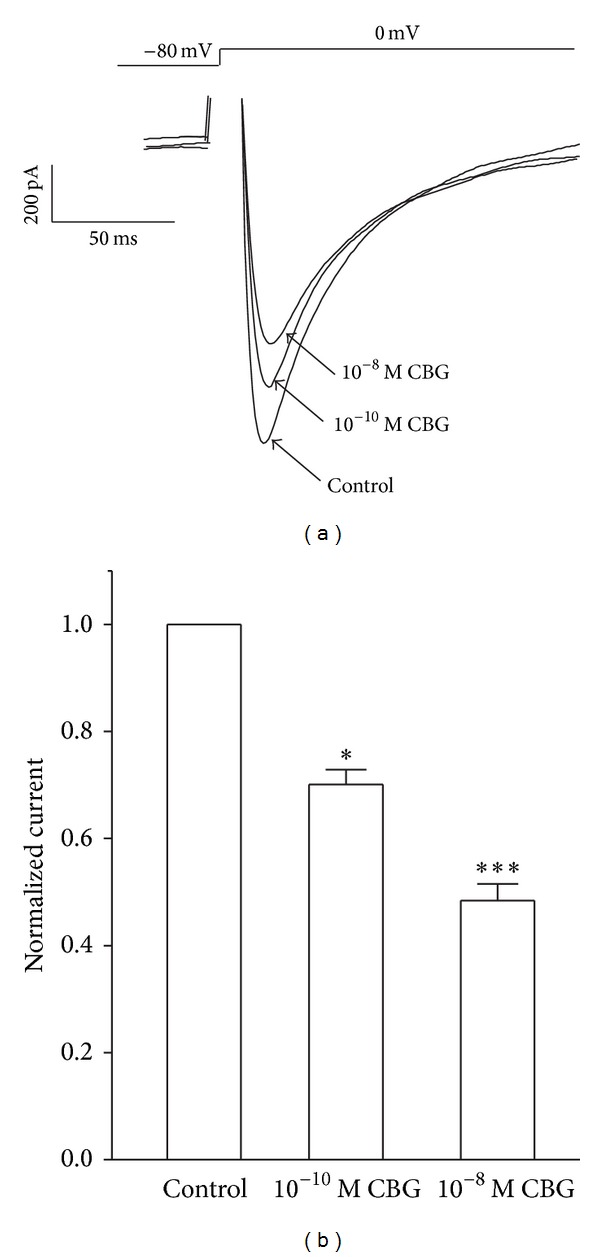
Effects of CBG on *I*_Ca-L_ of ventricular myocytes from rat hearts with myocardial ischemia. *I*_Ca-L_ was recorded under control conditions, during exposure to 10^−10^ and 10^−8^ M CBG. Data are means ± S.E.M. (*n* = 5 cells). *P < 0.05, ***P < 0.001, versus control.

Representative current recordings with the activation protocol after the sequential handles of 10^−10^ and 10^−8^ M CBG are shown in Figure 5. The peak amplitude of *I*_Ca-L_ was decreased by 29.99 ± 2.78% and 51.57 ± 3.12% by CBG derivatives at 10^−10^ and 10^−8^ M, respectively.

### 3.5. Effect of CBG on Steady-State Activation and Inactivation of *I*_Ca-L_

**Figure 6:**
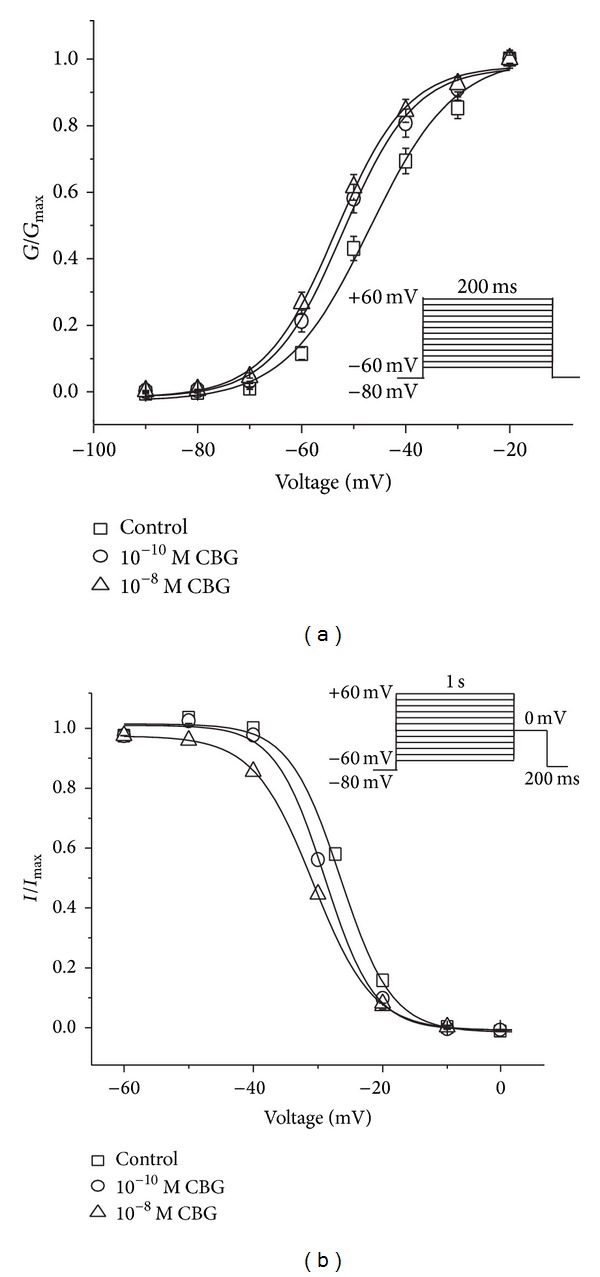
Effects of CBG on voltage-gated LTCCs in isolated rat left ventricular myocytes. (a) The steady-state activation of LTCCs shifted left by application of 10^−10^ and 10^−8^ M CBG. Tail currents were elicited by depolarization to −60 mV after 200 ms test pulses from −60 to 0 mV in increments of 10 mV. (b) CBG shifted the steady-state inactivation curve of LTCCs to the hyperpolarizing direction. The voltage protocol included double pulses consisting of a 200 ms test pulse to 0 mV following a 1 s conditioning pulse varying from −60 to 60 mV with 10 mV increments. Data are presented as means ± S.E.M. (*n* = 8 cells).

CBG caused a significant leftward (hyperpolarizing) shift in the voltage dependence of the *I*_Ca-L_ activation curve (Figure 6(a)). The* V*_1/2_ value for activation in the control was −46.90 ± 1.21 mV with a slope factor (*k*) of 7.95 ± 1.07 mV. Meanwhile,* V*_1/2_ values for activation with 10^−10^ and 10^−8^ M CBG were −52.02 ± 0.80 mV with a* k* value of 6.77 ± 0.71 mV and −53.44 ± 0.60 mV with a* k* value of 6.78 ± 0.55 mV, respectively, compared with the control group. As shown in Figure 6(b), CBG also caused a marked hyperpolarizing leftward shift in the voltage dependence of the *I*_Ca-L_ inactivation curve. The result shows the* V*_1/2_ value for inactivation was −26.5 ± 0.61 mV with a* k* value of 3.98 ± 0.45 mV. In the presence of 10^−10^ and 10^−8^ M CBG,* V*_1/2_ values for inactivation were −29.02 ± 0.50 mV with a* k* value of 3.82 ± 0.54 mV and −30.70 ± 0.10 mV with a* k* value of 4.60 ± 0.07 mV, respectively.

### 3.6. Effects of CBG on Cell Contraction and Ca^2+^ Transient in Rat Ventricular Myocytes

**Figure 7:**
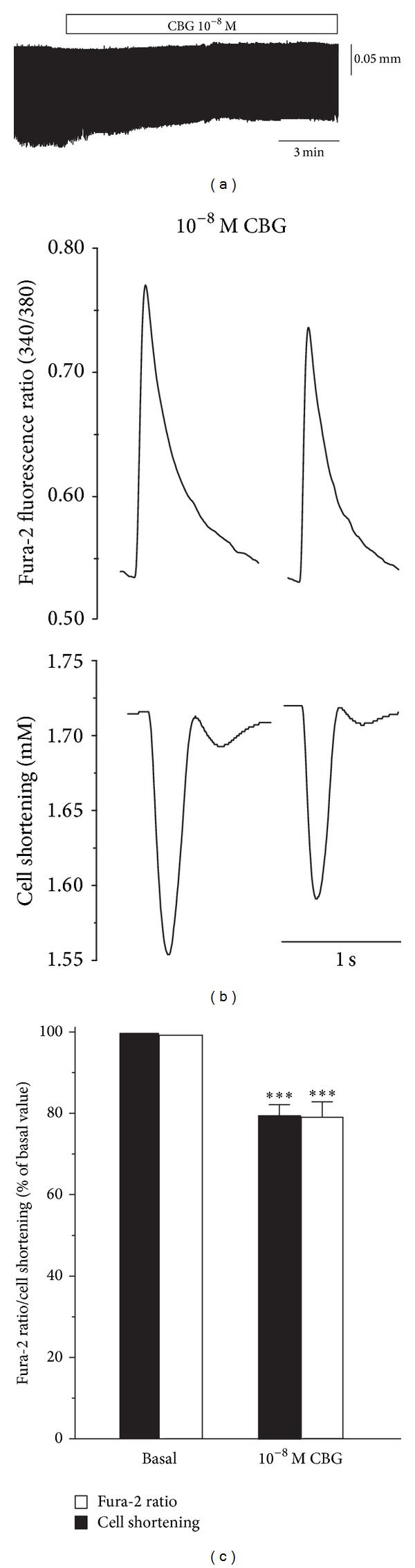
Effects of CBG on Ca^2+^ transients and cell shortening in isolated rat ventricular myocytes. (a) Actual tracings of effects of 10^−8^ M CBG in a myocyte. (b) Individual signals of fura-2 ratio (upper tracings) and cell shortening (lower tracings). Individual tracings were obtained by averaging five successive signals. (c) Summarized data on effects of 10^−8^ M CBG on fura-2 ratio and cell shortening. Data are presented as means ± S.E.M. (*n* = 7–12 cells). ***P < 0.001, compared with control.

Actual tracings of the effect of CBG (10^−8^ M) on cell shortening in ventricular myocytes are shown in Figure 7(a), and representative cell shortening and Ca^2+^ transients recordings before and after application of CBG (10^−8^ M) are provided in Figure 7(b). The results showed that CBG decreased the maximum cell shortening along with the decrease in Ca^2+^ transients. The extent of the CBG-induced decreases in cell shortening and Ca^2+^ transients was similar (Figure 7(c)), and an association between the amplitudes of cell shortening and Ca^2+^ transients was observed with CBG.

### 3.7. Effects of CBG on Cell Tp and Tr

**Figure 8:**
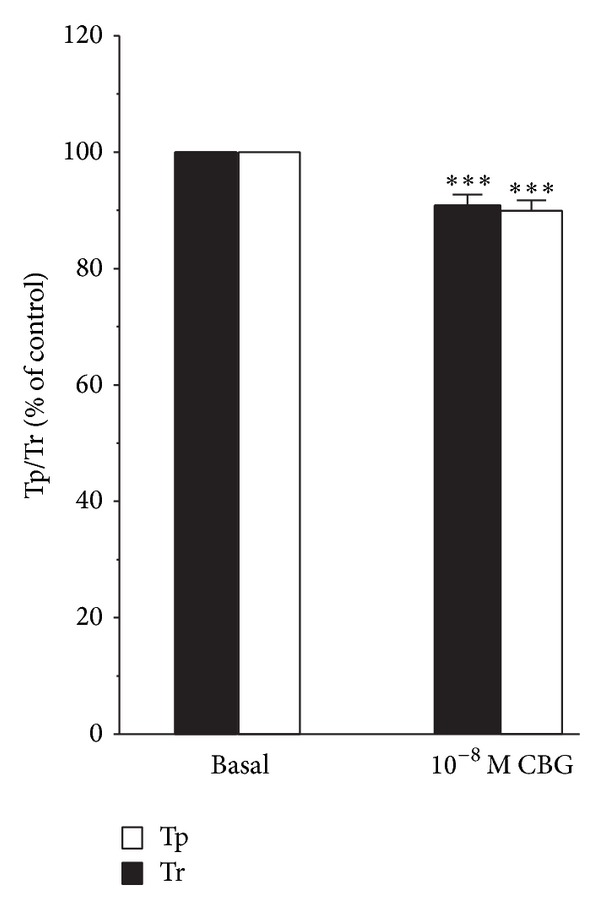
Summarized data for the effect of CBG on Tp and Tr for myocyte shortening. Changes in timing were measured in milliseconds and normalized to changes in the peak amplitude. Data are presented as means ± S.E.M. (*n* = 7–9 cells). ***P < 0.001, compared with control.

We also examined whether CBG causes changes in the timing of myocyte shortening and/or Ca^2+^ transients. The time to peak (Tp) is a characterization of the speed of contraction or Ca^2+^ elevation, while the time to 50% of the baseline (Tr) is a characterization of cellular relaxation or Ca^2+^ reuptake.  Figure 8 shows that CBG (10^−8^ M) decreased Tp and Tr for myocyte shortening. Only 7–9 cells could be obtained for analyzing the changes in Tp or Tr.

## 4. Discussion

It has been well established that some bufadienolides can attack multiple extracellular or intracellular targets, including voltage-gated Ca^2+^ channels [8] and K^+^ channels [2]. CBG was shown to act as an inhibitor of outward delayed-rectifier K^+^ currents with significant inhibitory effects on the kinetic properties of the K^+^ channel [16]. However, the involvement of CBG in the modulation of the Ca^2+^ channel activity in ventricular myocytes has not been reported. Our present study is the first to document the inhibitory effects of CBG on LTCC, Ca^2+^ transient, and contractility in rat ventricular myocytes. We further examined the physiological role of CBG, as summarized below.

Ca^2+^ channels are found in all excitable cells and are essential for electrical excitability, excitation-contraction coupling, excitation-secretion coupling, and other cellular functions [22]. The development of drugs that block Ca^2+^ channels has provided a valuable route for studying channel function, and clinical use of Ca^2+^ channel blockers in combating CHD and Ca^2+^ overload is rapidly increasing. Here, we studied the effects of CBG on ventricular myocytes. Our results showed that CBG could induce certain electrophysiological changes in the properties of *I*_Ca-L_, including blocking of peak current, shifting of activation and inactivation curves, and acceleration of attenuation. The peak of *I*_Ca-L_ was decreased by CBG in a concentration-dependent manner in rat normal ventricular myocytes (Figure 3) and was decreased by CBG with 10^−10^ and 10^−8^ M in rat ischemic ventricular myocytes (Figure 5). The steady-state activation and inactivation curves were left-shifted (Figure 6), and the* I-V* curve was shifted upward (Figure 4(b)). The influence of CBG on these parameters may help to explain the mechanisms of its effects on Ca^2+^ channels. Hence, it is reasonable to infer that CBG exerts its protection on cardiomyocytes at least partly by inhibiting *I*_Ca-L_. Furthermore, we also wanted to know whether this effect of CBG was due to interference at multiple sites or the resultant imbalance in ionic homeostasis and Ca^2+^ metabolism could be attributed to other reasons.

Ca^2+^ is a critical intracellular factor in excitatory-contraction coupling [23]. The [Ca^2+^]_i_ transient elicited by electrical field stimulation is known to depend on the influx of Ca^2+^ via the LTCC and Ca^2+^ release from the sarcoplasmic reticulum (SR) induced by the entering Ca^2+^ [24]. The opening of voltage-dependent LTCCs induced by excitation of cardiomyocytes facilitates Ca^2+^ flow into the cells, subsequently leading to an increase in [Ca^2+^]_i_. Abundant Ca^2+^ is released into cardiomyocytes within a short period of time from inner stores in the SR via Ca^2+^-induced Ca^2+^ release. This instantaneous rise in Ca^2+^ consequently activates the Ca^2+^ pump in the SR and Na^+^-Ca^2+^ exchanger in the cytomembrane. Finally, the Ca^2+^ pump and Na^+^-Ca^2+^ exchanger transport the Ca^2+^ into the SR and out of cardiomyocytes, and the [Ca^2+^]_i_ decays immediately thereafter. This phenomenon is termed the Ca^2+^ transient [25].

There is a good correlation between voltage dependence of the *I*_Ca_ and that of Ca^2+^ transient. Ca^2+^ transient is inhibited when the *I*_Ca_ is blocked by the addition of LTCC antagonists, despite the fact that SR still contains Ca^2+^ that can be released by caffeine (with inhibition of this release by ryanodine). The cause of the increased Ca^2+^ release from the SR is that an increase in *I*_Ca_ triggers the release of more Ca^2+^ from the SR and inhibition of *I*_Ca_ by LTCC antagonists resulted in a decrease in the amplitude of the Ca^2+^ transient, presumably by decreasing the Ca^2+^ load of the SR. There is good evidence that SR Ca^2+^ release can be “graded” by the amount of trigger Ca^2+^ entering the cell [26].

In this study, treatment of cardiomyocytes with CBG (10^−8^ M) decreased the Ca^2+^ transients (Figure 6). The results indicated that CBG may have indirectly restrained Ca^2+^ release from the SR possibly through inhibition of the activity of the *I*_Ca-L_ channel (Figure 3), thus reducing free [Ca^2+^]_i_. CBG at 10^−8^ M also was shown to cause a decrease in Tp and Tr for myocyte shortening (Figure 8). In fact, the contraction reaction depends on the concentration of free [Ca^2+^]_i_ [27]. Moreover, cardiac muscle is highly dependent upon the normal function of Ca^2+^ influx, and excitation-contraction coupling in cardiac cells requires Ca^2+^ influx. Therefore, inhibitory effects of CBG on LTCC can result in reduced cardiac contractility. In this study, CBG restrained the *I*_Ca-L_ and thus reduced free [Ca^2+^]_i_, ultimately leading to a decreased amplitude of the ventricular myocyte contraction (Figure 7) and the protection of the cardiac cell. This reduction of contractility and consequently the decrease of myocardial oxygen consumption appear to be an important cellular mechanism targeted for clinical treatment of myocardial ischemia [27].

An additional effect of LTCC blockers has been demonstrated in ischemic cardiomyopathy. Because ischemia causes membrane depolarization, Ca^2+^ influx in ischemic cells is increased. Elevated [Ca^2+^]_i_ accelerates the activity of several ATP-consuming enzymes, which further depletes already marginal cellular energy stores and renders the heart even more susceptible to ischemic damage [28]. The benefits of Ca^2+^ channel blockers have been proven to include protection against the damaging impacts of Ca^2+^ by reducing the incidence of arrhythmia and ultimate size of developing infarctions in experimental animals [28]. On the other hand, the persistent increases in Ca^2+^ influx through LTCCs lead to apoptosis through a mitochondrial death pathway [29]. Apoptosis is a critical component of myocyte death in congestive heart failure and after myocardial infarction, persistent hemodynamic stress, and aging [30]. Therefore, CBG exerts its beneficial effect by reducing Ca^2+^ influx and lowering [Ca^2+^]_i_ indirectly.

However, previous studies on CBG generally focused on its cardiotoxicity. CBG and other bufanolide steroid compounds have been reported to have similar chemical structures to digitoxigenin (Figure 1) and to possess both pharmacological and toxicological effects in* in vitro* and* in vivo* studies. The lower concentrations (10^−12^, 10^−11^, 10^−10^, 10^−9^, and 10^−8^ M) of CBG tested in our current study all fall into a safe range, where the LTCC remains intact and functioning in the reduction of cardiac [Ca^2+^]_i_ (Figures 2 and 7). This finding is different from simply determining the cardiotoxicity. Effects of CBG on *I*_Ca-L_, Ca^2+^ transient, and contraction in rat ventricular myocytes also were all observed within the range of safe concentrations.

In summary, CBG was demonstrated to have significant inhibitory effects on *I*_Ca-L_ in a concentration-dependent manner and to reduce Ca^2+^ transients and cell contraction in ventricular myocytes. The results reasonably suggest that the inhibitory effects of CBG may have therapeutic benefits and support a possible link between the cardioprotective effects of CBG and Ca^2+^ channels. The findings also provide experimental evidence for rational applications of Kyushin and SBP for patients in the clinic. Because the CBG-mediated mechanism of *I*_Ca-L_ suppression was not directly determined in the present study, further work is necessary to better understand the precise mechanism for the inhibitory effects of CBG on *I*_Ca-L_.

## Abbreviations

*I*_Ca-L_: L-type Ca^2+^ current
LTCC: L-type Ca^2+^ channel
KB: Kreb's buffer
CHD: Coronary heart disease
SBP: Shexiang Baoxin Pill
MI: Myocardial infarction
SR: Sarcoplasmic reticulum
Tp: Time to peak
Tr: Time to 10% recovery
HPLC: High performance liquid chromatography.
